# A Computational Model of Innate Directional Selectivity Refined by Visual Experience

**DOI:** 10.1038/srep12553

**Published:** 2015-07-31

**Authors:** Samantha V. Adams, Christopher M. Harris

**Affiliations:** 1 Centre for Robotics and Neural Systems, University of Plymouth, PL4 8AA Plymouth, United Kingdom

## Abstract

The mammalian visual system has been extensively studied since Hubel and Wiesel’s work on cortical feature maps in the 1960s. Feature maps representing the cortical neurons’ ocular dominance, orientation and direction preferences have been well explored experimentally and computationally. The predominant view has been that direction selectivity (DS) in particular, is a feature entirely dependent upon visual experience and as such does not exist prior to eye opening (EO). However, recent experimental work has shown that there is in fact a DS bias already present at EO. In the current work we use a computational model to reproduce the main results of this experimental work and show that the DS bias present at EO could arise purely from the cortical architecture without any explicit coding for DS and prior to any self-organising process facilitated by spontaneous activity or training. We explore how this latent DS (and its corresponding cortical map) is refined by training and that the time-course of development exhibits similar features to those seen in the experimental study. In particular we show that the specific cortical connectivity or ‘proto-architecture’ is required for DS to mature rapidly and correctly with visual experience.

Aspects of the mammalian visual system such as retinotopy [point-to-point topographic connections between the retina, lateral geniculate nucleus (LGN), and cortical visual area 1 (V1)], and cortical maps for ocular dominance (OD), orientation (OR) and directional selectivity (DS) have all been well studied experimentally. In some species (such as ferrets, cats, and primates) cortical maps for OR and DS possess a distinct patchy structure with features such as pinwheels, saddle points and discontinuities^1^,^2^,^3^. In these species, DS appears to arise first in visual cortex and depends on cortical developmental plasticity after eye-opening (EO)^4^, but exactly how this occurs and the importance of pre-EO structures are poorly understood.

Earlier studies showed that significant OR maps were already present at EO, but that DS maps developed post-EO with visual experience^5^,^6^. However, a weak DS bias has been recently reported at EO in ferret, which then develops rapidly with visual experience during the first few weeks^7^,^8^. The implication is that DS may be present prior to visual experience^9^. However, whether this precocious DS is activity dependent is unclear. There is some experimental evidence to suggest that pre-EO neural activity (retinal waves) may be responsible^4^,^7^, and this has also been investigated in several computational studies^10^,^11^,^12^,^13^. It is also possible that merely testing for DS is sufficient to develop DS in an incipient network. A third possibility is that DS exists independent of neural activity and is laid down by physical growth of a neural ‘proto-architecture’ in the form of retino-LGN-V1 network connections. Such a proto-architecture could potentiate rapid learning once visual experience becomes available.

In a recent modelling study, we proposed an artificial spiking neural network model to investigate how its structure and connectivity could provide an initial DS capability prior to visual experience^14^ (Fig. 1). The advantage of a modelling approach is that we could switch off the network’s learning ability to examine DS which is not possible *in-vivo*. We found that a basic cortical structure with ‘Mexican hat’ connectivity, distance-dependent delays, and afferent input layer with ‘receptive fields’ produced DS and OR maps similar to those seen experimentally (and in previous modelling studies).

We concluded that this ‘innate’ structure was sufficient to produce DS prior to any visual experience. Experimental findings from ferret have shown that DS at EO was stronger in layer 4 of V1 than layer 2/3, and that layer 4 DS developed more rapidly up to post-natal day (PND) 35^8^. Therefore, as it seems likely that our prospective proto-architecture for DS resides in layer 4 of V1, in the current work we specifically compare our results with learning to those found for layer 4.

Given this functionally plausible model, we now ask two fundamental questions. First, does this proto-architecture learn from visual experience in the same way that real networks do? That is, is there refinement of the patchy DS map with training, or is the patchiness lost? To test this, we employed spike timing dependent plasticity (STDP) as a biologically plausible learning rule. We show that the major features of the natural developmental time course of DS and OR are reproduced and we investigate how this plasticity operates to cause refinement of DS.

Second, does a proto-architecture provide any selective advantage to the organism, such as facilitating learning after EO? We show that ‘lesioning’ the model disrupts the ability to learn from visual experience, and that an initial structure is essential for rapid learning. In conclusion, we propose that for mammals that develop patchy cortical DS and OR maps, a proto-architecture is likely to be an essential component.

## Results

### Visual experience sharpens DS and OR selectivity

We used Spike-Timing Dependent Plasticity (STDP) (see Learning subsection in Methods) on all the connections in the model. We generated ten randomly initialised networks and trained them with 50 random presentations of a bar object moving in one of eight different directions (replicating the ‘visual experience’ of real animals after EO). Before switching on learning, the initial DS preference map was patchy (Fig. 2a). After switching learning on, the patchiness was not lost but became more delineated with sharper boundaries (Fig. 2b,c). As training progressed the maps developed characteristics seen in experimentally derived maps such as areas of smoothly changing preference, fracture lines and saddle points^1^,^2^. To quantify the strength of preference, we employed a ‘selectivity index’ (SI) for neurons in the networks at various stages of training using a vector average method (see Supplementary Methods). SI takes on values between 0.0 (no preference) and 1.0 (exclusive preference for one direction or orientation). The average DS SI increased with training from 0.1 to 0.32, and compares favourably with experimental measurements of average DS SI in ferret of 0.12 at EO to 0.30 at postnatal day (PND) ≥ 35^8^. OR preference maps showed a similar pattern of development. Average OR SI increased with training from 0.21 to 0.36 compared with experimental measurements in ferret of 0.24 at EO to 0.48 at PND ≥ 35^8^. Figure 3a,b) (top) shows cumulative percentage curves of SI calculated for all neurons over all runs for the initial (EO) condition (grey dashed line) and the trained condition (solid black line). Figure 3c,d) (bottom) shows the experimental curves^8^ for layer 4.

Our computationally derived curves are similar to the experimental ones. The initial curves show distinct ‘corner’ shapes (in particular DS) shifted to the left reflecting the low average SIs. Training increased the selectivity strength for both DS and OR considerably causing a distinctive rightwards shift and a more S shaped curve exactly as seen in^8^ (Mann-Whitney test, α = 0.05, n = 36000, p < 2.2e-16).

We also investigated how responses to the preferred and null directions and orthogonal orientation changed over time compared to experimental data^8^. The preferred direction is the direction for which the neuron has the largest response (number of spikes generated during a pattern presentation), the null direction is the opposite direction to the preferred and the orthogonal orientation is at 90 degrees to the preferred/null directions. We collected responses to all directions for all neurons in one run, and to enable comparisons for all neurons we shifted the data so that the preferred direction response was always aligned at 0 degrees for all neurons and therefore the null direction and orthogonal orientation responses also aligned at 90 and 180 degrees respectively. Any neurons which did not respond to any direction were discarded. We performed Mann-Whitney tests for three specific comparisons (with adjusted alpha of 0.0167 for 95% confidence level after a Bonferroni correction): comparing changes in response to preferred direction in the initial and midway cases (n = 3285, 3319, average response increased from 0.23 to 0.34, p < 2.2e-16) ; to the null direction in the midway and final cases (n = 3285, 3352, average response decreased from 0.16 to 0.12, p < 2.2e-16) and to the orthogonal orientation in the midway and final cases (n = 3285,3352, average response decreased from 0.08 to 0.07, p < 2.2e-16). Figure 4 shows the average responses to all directions for the initial, midway and final cases. To compare with^8^, we fitted the three cases separately with a double Gaussian function, with the preferred mean set at 0 degrees, and the null mean at 180 degrees. The standard deviations of the preferred and null Gaussians were constrained to be the same, and found using the least-squares method. As training progressed, the responses to preferred and null directions change from being roughly equal to a situation where the preferred response was much larger, indicating that the neurons had developed DS. These results are in agreement with those of^8^ who found that the response to the preferred direction increased in the first half of training (EO to PND < 35) and the responses to the null direction and orthogonal orientation decreased in the second half of training (PND < 35 to PND >= 35). Our Fig. 4 closely resembles the experimentally derived tuning curves in Fig. 2 from^8^.

### Disruption of initial afferent and lateral connectivity prevents training from refining OR and DS

In order to further investigate the importance of the network structure in delivering an early DS capability, we examined the effect of disruptions to the initial structure on the ability to develop mature DS. In our previous work we examined how changing afferent and lateral connectivity from the default case affected the proto-architecture’s innate ability to represent DS and OR in the ‘EO’ condition^14^. We found that the worst disruption occurred when there was no receptive field structure in the afferent connections (i.e. the LGN and Cortical layers were fully connected) and when the extent of lateral inhibition in the Cortical layer was reduced. In the current work we investigated whether the same connectivity disruptions affected the network’s ability to refine with visual experience or in fact capability is restored by training. We performed learning experiments with both full LGN-Cortical connectivity and the spatial range of lateral inhibition drastically reduced. This was done by forcing the lateral inhibition to zero at distances greater than eight units, effectively reducing the range from approximately five times the excitatory range down to only two times. All other network parameters (e.g. starting weights) were kept the same.

Five randomly initialised networks were generated with disrupted afferent and lateral inhibitory connectivity and trained in the same way as for the intact case. Initially, selectivity was so low it was impossible to assign strong preferences to neurons hence the complete lack of features (Fig. 2d). As training progressed through mid-training (Fig. 2e) to the final state (Fig. 2f), individual neurons did acquire preferences but no coherent map emerged. Average SI strength increased for DS from 0.02 to 0.12 and for OR from 0.09 to 0.11, but final values were much weaker compared to the intact case. The impact of connectivity disruption on training was very clear in the cumulative percentage curves (Fig. 5). The initial distributions (grey dashed line) comprised of only very low SI values, and the trained curves (solid black line) showed some shift to higher Sis but only for low strengths. The cumulative percentage distributions seen in the intact connectivity case and in the experimental data (Fig. 3a–d) were absent.

### Uni and bi – directional training alter initial DS biases in favour of the trained directions

Experimental studies have examined the extent to which the initial DS bias in ferret could be overridden by visual experience with either training in only one direction^7^, or with two directions only (same orientation)^6^. For comparison, we performed the same training with our network. For the uni-directional case, we trained only with stimuli moving left to right (direction E) and found that the number of neurons that acquired a preference for this direction (with a tolerance of +/−5°) increased (n = 154) compared to the case when trained with all eight directions (n = 133). We also found that the average DS selectivity index (SI) after training was larger for the neurons which had a preference for the trained direction (SI = 0.45) compared to all other neurons (SI = 0.23) (Mann-Whitney, p < 2.2e-16). It should be noted, however, that all neurons increased their DS SI significantly from the initial untrained case where average SI was 0.09. These results mainly agree with experiment, except that we found that training was very effective in changing neurons’ initial preference regardless of the strength or direction of that bias, whereas^7^ observed that neurons with an initial strong bias opposite the trained direction did not change their bias.

For bi-directional training we used stimuli moving rightward (direction E) and leftward (direction W) and ensured there was approximately an equal number of presentations of each direction. The number of neurons that acquired a preference for the E direction (+/−5°) increased (n = 141) compared to the case when trained with all eight directions (n = 133). The number of neurons that acquired a preference for the W direction (+/−5°) decreased (n = 113) compared to the case when trained with all eight directions (n = 149). We also found that the average DS selectivity index (SI) after training was larger for the neurons which had a preference for one of the trained directions. For direction E, SI = 0.22 compared to non-trained directions SI = 0.21 (Mann Whitney, p = 0.01657). For direction W, SI = 0.28 compared to non-trained directions SI = 0.21 (Mann Whitney, p = 0.000655).

### Weight increases on Cortico-Cortical connections are the primary driver for refinement of DS

To investigate in more detail how the plasticity method we used caused refinement of DS with visual experience we firstly examined weight changes on the afferent (LGN-Cortical) and lateral (Cortico-Cortical) connections after uni-directional training. We selected neurons which acquired a preference for the trained direction (moving left to right) with low, medium and high SI values and measured the change in connection weights. Figure 6a (left) shows plots of LGN-Cortical weight (initial-final) for three example neurons. We saw a clear asymmetry in the weight changes related to the training direction - generally there are weight increases on the left and decreases on the right on all three plots in Fig. 6a. We also found that the asymmetry was more pronounced as SI increased. Figure 6b (right) gives an example of lateral excitatory and inhibitory weight changes typical for all neurons examined. Excitatory and inhibitory lateral weights strengthened during the course of training but we saw no asymmetry related to the training direction, however, larger weight increases occurred on synapses to neurons with larger SI values. These results implied that plasticity on both afferent and cortical connections contributed to the development of DS.

To further clarify the relative roles of afferent and lateral weight changes we firstly repeated the uni-directional training experiment, disabling afferent and lateral plasticity in turn. Secondly, to examine the impacts on SI distribution and DS map structure we performed experiments training with all eight patterns with plasticity disabled on the afferent and lateral connections in turn. We found that with cortical plasticity disabled, there was no significant refinement of DS. For the uni-directional training, average SI for the neurons which had a preference for the trained direction increased slightly (SI = 0.11) compared to other neurons (SI = 0.09), but this was not statistically significant (Mann-Whitney, p = 0.5921). Examples of afferent weight changes without cortical plasticity are given in Fig. 7a. These weights show asymmetry in parts but are not as smoothly varying and do not show the relationship to the training direction or SI as seen in Fig. 6a. Figure 8a,b show the cumulative percentage graph and DS map extract for training with all patterns with cortical plasticity disabled. The average SI, distribution of SI and the DS map are very similar to those from the untrained state (see Fig. 2a and Fig. 3a, grey dashed line for comparison). With only afferent plasticity disabled, refinement of DS occurred normally. For the uni-directional training, average SI for neurons that acquired a preference for the trained direction increased (SI = 0.45) compared to other neurons (SI = 0.23) and was statistically significant (Mann-Whitney, p < 2.2e-16), so a very similar result to training with both afferent and cortical plasticity enabled. Figure 7b shows an example of typical excitatory and inhibitory cortical weight changes. Weights increased on both excitatory and inhibitory connections in a similar manner to when both afferent and lateral plasticity were enabled. Again we saw no asymmetry particularly related to the training direction. Figure 8c,d show the cumulative percentage graph and DS map extract for training with all patterns with afferent plasticity disabled. The average SI, distribution of SI and the DS map were very similar to the trained state (see Fig. 2c and Fig. 3a, solid line for comparison). These results imply that, in our model, cortical plasticity is in fact the main driver for refinement of DS. Cortical plasticity seems to contribute somewhat to weight changes on the afferent connections as the asymmetry related to the training direction was more pronounced when cortical plasticity was enabled.

## Discussion

**Figure 1:**
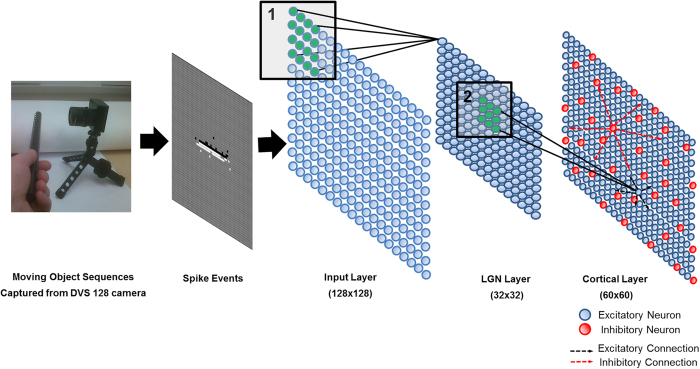
Overview of the network architecture. Moving left to right across the figure, input patterns are generated by moving bar-like objects in front of the DVS camera. This generates a 128 × 128 sized array of spike events (black and white pixels). These are applied directly to the Input layer which is also 128 × 128. The connections from the Input layer to the LGN layer are such that sixteen neurons (shown in Box 1) in the Input layer are fed into one neuron in the LGN layer thus achieving a down-sampling to 32 × 32. The LGN layer is connected to the Cortical layer with overlapping connection fields (neurons shown in Box 2) so that each cortical neuron sees only a portion of the LGN. The cortical layer has recurrent connections in a Mexican hat arrangement (short range excitatory connections between neighbouring neurons and longer range inhibitory connections between more distant neurons).

**Figure 2:**
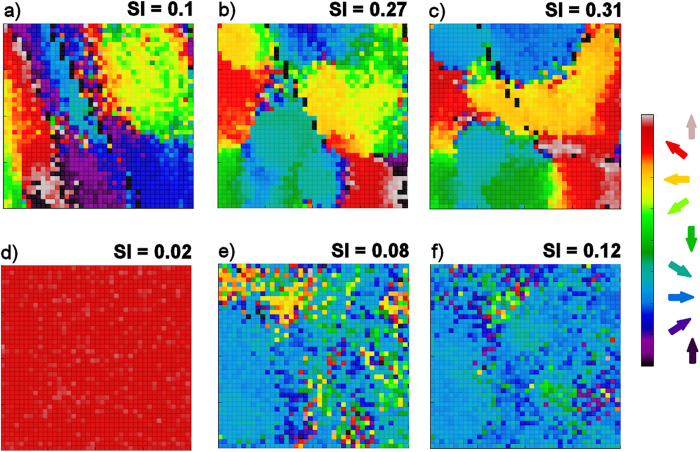
Extracts from direction preference maps. Intact connectivity conditions: (**a**) Initial/ at EO, (**b**) mid-training, (**c**) after training. Disrupted connectivity conditions: (**d**) Initial/ at EO, (**e**) mid-training, (**f**) after training. We created direction preference maps using a standard vector average method (described more fully in Supplementary Methods) and extracted the same rectangular area for each. Numbers indicate the average selectivity (SI). (**a**) shows how in the intact connectivity case the initial map is patchy and there are some areas of smoothly varying preference but the map is immature and does not have all the features seen in mature maps. (**b**,**c**) show the effects of training to refine the selectivity and the arrangement of preferences to form a smoothly varying map. In (**d**) the initial case for disrupted connectivity, there is no evident map structure and selectivity is so low that distinct preferences cannot be detected. (**e**,**f**) with training, many neurons acquire a preference for one of the directions as shown by the speckling in the map. However, there is no true map structure with patches of similar preference smoothly varying across the cortical area as in (**b**) and (**c**).

**Figure 3:**
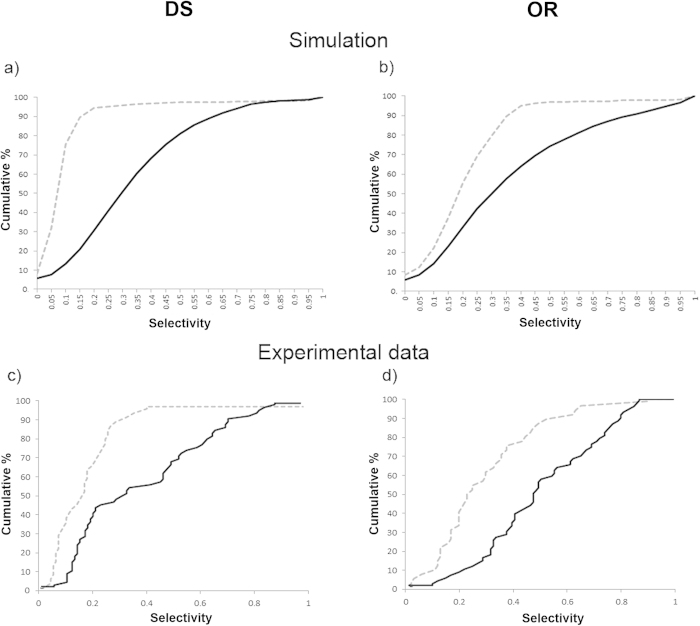
Intact connectivity: cumulative % curves of selectivity (SI) for (**a**) DS and (**b**) OR generated from our computational model and (**c**) DS and (**d**) OR generated from experimental data for layer 4 (same data as shown in Figs 3c and 4c from^8^). In (**a**) and (**b**) the Selectivity Index (SI) for both DS and OR has been calculated for all neurons from ten randomly initialised networks both before and after training and cumulative percentage curves calculated to show the distributions. (**a**) distributions for DS for the initial (grey dashed line) and trained (solid black line) cases. (**b**) distributions for OR for the initial (grey dashed line) and trained (solid black line) cases. In both cases the effect of training is to shift the curve rightwards and increase the average selectivity. (**c**) DS for layer 4 neurons at EO (grey dashed line) and at PND ≥ 35 (solid black line). (**d**) OR for layer 4 neurons at EO (grey dashed line) and at PND ≥ 35 (solid black line). With visual experience the average selectivity for both DS and OR increases. The distribution curves shift to the right maintaining a proportion of low and high responders.

**Figure 4:**
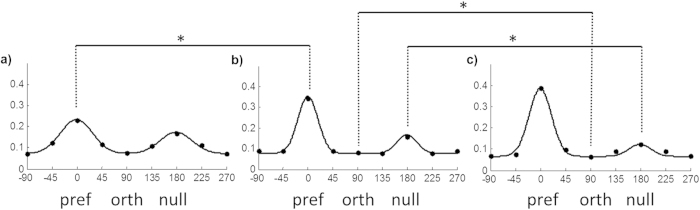
Changes in response to null and preferred direction and orthogonal orientation during training. Responses (y axis) are normalised rate. Raw data points are overlaid with fitted double Gaussians with the preferred response fixed at 0. (**a**) Initial case before training, (**b**) midpoint of training and (**c**) at the end of training. As training progressed the responses to preferred and null directions change from being roughly equal to a situation where the preferred response is much larger indicating the development of DS. In agreement with the experimental results of^8^, the response to the preferred direction (pref) increases greatly in the first half of training. The responses to the null direction (null) and orthogonal orientation (orth) decrease during the second half of training. *indicates differences across conditions that were significant with Mann-Whitney pairwise tests.

**Figure 5:**
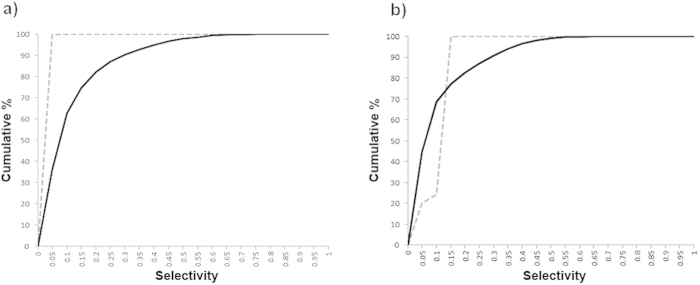
Disrupted Connectivity: cumulative % curves of selectivity (SI) for (**a**) DS and (**b**) OR for untrained (grey dashed line) and trained (solid black line) cases. The Selectivity Index (SI) for both DS and OR has been calculated for all neurons for five randomly initialised networks with disrupted connectivity both before and after training and cumulative percentage curves calculated to show the distributions. (**a**) distributions for DS for the initial (grey dashed) and trained (solid black) cases. (**b**) distributions for OR for the initial (grey dashed) and trained (solid black) cases. In both cases the initial curves are different to those for the intact case in Figs. 3a and b and training does not shift the curves rightwards or increase selectivity by a large amount.

**Figure 6:**
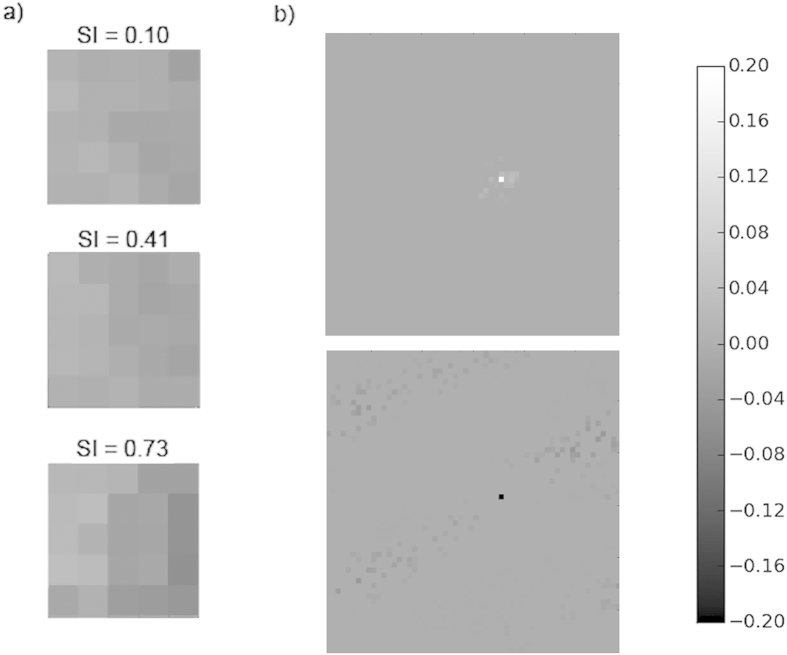
Effects of uni-directional training on neurons’ afferent and lateral connection weights, (final – initial) weights are plotted in all cases. (**a**) Changes in afferent (LGN-Cortical) weights for neurons of different SI (**b**) Typical changes in lateral excitatory and inhibitory weights (solid black and white pixels indicate the location of the neuron in question). Following training only with stimuli moving left to right afferent connections show asymmetric weight changes in keeping with this direction and the asymmetry is stronger with stronger SI. Lateral weight changes do not show asymmetry related to the training direction but there is strengthening in both short range excitatory connections and longer range inhibitory connections.

**Figure 7:**
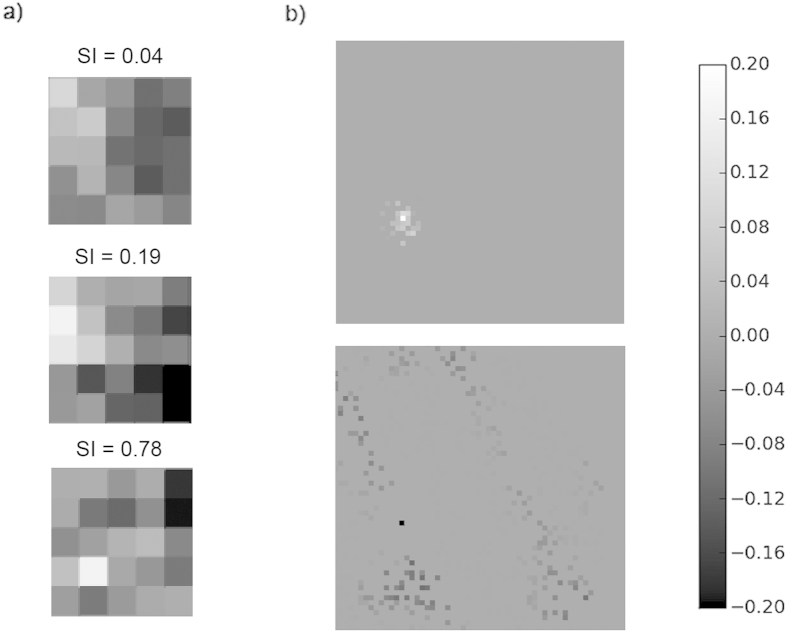
Effects of uni-directional training on neurons’ afferent and lateral connection weights when either afferent or cortical plasticity was disabled, (final – initial) weights are plotted in all cases. (**a**) Changes in afferent (LGN-Cortical) weights for neurons of different SI when cortical plasticity was disabled (**b**) Typical changes in lateral excitatory and inhibitory weights when afferent plasticity was disabled (solid black and white pixels indicate the location of the neuron in question). When cortical plasticity was disabled and the network trained only with stimuli moving left to right, afferent connections show changes but these do not obviously match the training direction and there is no particular relationship to SI. With afferent plasticity disabled, lateral weight changes were similar to when both afferent and cortical plasticity were enabled: they do not show asymmetry related to the training direction but there is strengthening in both short range excitatory connections and longer range inhibitory connections.

**Figure 8:**
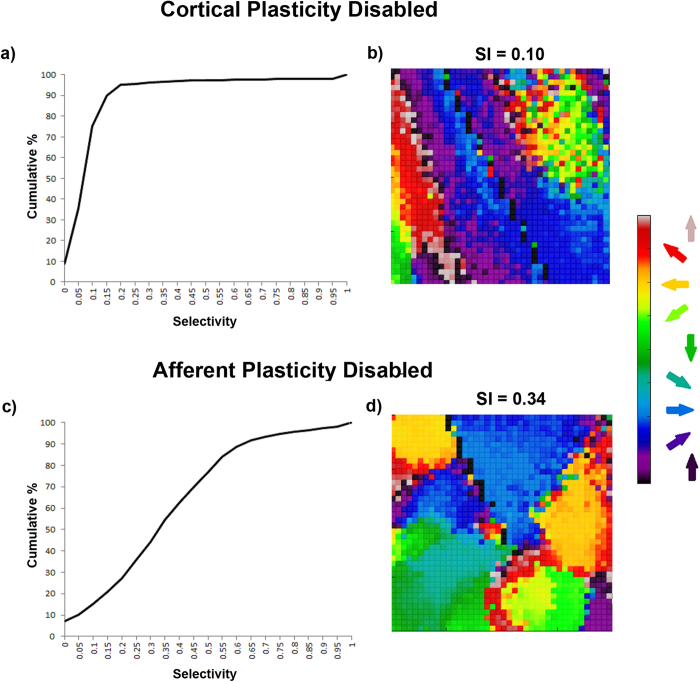
Cumulative percentage curves and extracts from the DS map for training when either afferent or cortical plasticity was disabled. (**a**,**b**)—cortical plasticity disabled (**c**,**d**)—afferent plasticity disabled. When cortical plasticity was disabled, refinement of the DS map and increase in SI did not occur. The cumulative percentage curve in (**a**) looks similar to the untrained case shown in Fig. 3a (grey dashed line). The DS map in (**b**) looks similar to that of Fig. 2a. When afferent plasticity was disabled, refinement of the DS map and increase in SI proceeded normally. The cumulative percentage curve in (**c**) looks similar to the trained case shown in Fig. 3a (solid line). The DS map in (**d**) looks similar to that of Fig. 2c.

Our simple computational model (Fig. 1) generates patchy preference maps of directional selectivity prior to any training (Fig. 2a). This was observed by switching off any weight updating (learning) during testing, which is not possible in natural experiments. Our data closely match the experimental distributions for DS and OR in layer 4 of ferret V1 at EO^8^, implying that the model is sufficient to capture important features of the connectivity in layer 4 of V1. It also shows that the initial DS bias observed experimentally at EO^8^ may indeed arise from the structure of the network prior to any visual experience, rather than an artefact of rapid learning during testing.

For these initial maps to emerge, afferent receptive fields and lateral cortical connections (excitatory and inhibitory) were necessary. We found that lateral cortical inhibition was crucial, which is in agreement with experimental evidence that cortical inhibition plays an important part in the development of OR and DS^15^,^16^,^17^.

After switching on learning, we observed that exposure to visual input increased the degree of selectivity in DS and OR maps with sharper delineation among patches, but also with a distinctive spread of selectivity indicating that many neurons increased their selectivity, such that a distribution of neurons with low and high selectivity emerged as in^8^. This learning was remarkably rapid needing only 25–50 presentations of a moving object, which is a very small amount of training compared to typical computational studies (for example, 6000 presentations in^10^ and 20,000 image presentations in^12^). In the ferret, training has also been observed to be very rapid after EO^8^ – so much so that the experimental procedure for measuring the DS and OR of neurons needed to account for any training effect caused during measurement.

We previously disrupted the initial connectivity in various ways to explore which key features contributed to innate DS (structured afferent receptive fields and sufficient lateral inhibition in the Cortical layer) and in the current work have assessed the impact of these same disruptions on learning. We found that initial DS/OR selectivity was much weaker than with intact connectivity, and with learning switched on, recovery did not occur. We gave one of our disrupted networks double the amount of training and still saw no improvement, showing that a permanent impairment was caused by the disrupted connectivity. In particular the distributions of DS/OR selectivity were markedly different and we did not see the formation of patchy cortical maps.

In order to explore the plasticity mechanism further we performed experiments training with uni- and bi-directional stimulus motion and disabling either afferent or cortical plasticity. For uni-directional training, DS SI increased more for neurons with a preference for the training direction, as seen experimentally^7^. More neurons were converted to the training direction than when trained with all eight directions, as also seen experimentally^7^. We also found that training was able to completely override the initial DS biases of neurons, which contrasts with experimental data^7^ where neurons with an initial bias opposite to the training direction were less likely to be converted. For the bi-directional training we found that DS SI increased for both directions, as seen experimentally^6^ but the numbers of neurons converted only increased for one of the directions. A possible reason for these discrepancies is that only layer 2/3 neurons were examined experimentally^6^,^7^ and it is possible that the time-course or mechanism of plasticity may be different in layer 4. Following the uni-directional training experiment, we analysed the weight changes on both afferent and lateral connections for neurons which acquired a preference for the trained direction. For the afferent connections, asymmetric receptive fields developed after training with one direction of motion and also the asymmetry increased with DS SI. Increases in both lateral excitatory and inhibitory connections were observed, and were larger for neurons with larger SI but they were not asymmetric. From further experiments disabling either afferent or lateral plasticity we found that cortical plasticity alone was necessary and sufficient to explain the refinement of DS in our network (in terms of average SI, distribution of SI and changes in map structure). These results imply that initial weak DS in V1 could arise purely from the cortical connectivity and become stronger through strengthening of weights on only the cortico-cortical connections. The role of afferent connections in DS is unclear. From our previous work it appears they contribute something to the initial capability for DS^14^ but the current work we did not find that plasticity on afferent connections was required to replicate the refinement of DS seen in experiments.

Our model appears to be a minimal model inasmuch as it contains necessary and sufficient connectivity to replicate the observed experimental distributions of OR and DS at EO. With training, selectivity refined and distinctive maps with a smoothly varying patchy structure emerged, and various ‘lesions’ of the initial connectivity led to failure. We have not modelled how this proto-architecture itself grows. Presumably it results from a complex process of cellular replication and migration, steering of axonal and dendritic growth, and excitatory and inhibitory synaptogenesis that is ultimately genetically controlled. Such processes have been explored in computational studies^18^,^19^,^20^. Clearly, the output of this pre-EO process greatly influences the post-EO plastic development of DS and OR maps, and hence the long-term phenotype. How variation in this growth impacts on post EO function remains to be explored. We also have not yet considered the role of structural plasticity (synaptogenesis and pruning) and critical periods but intend to do so in future work.

As we have shown here, computational modelling of biological neural networks can be an important tool to complement and supplement experiments in developmental plasticity. With computational models, it is possible to do things that cannot be done experimentally – for example run many more trials, sample more neurons, experiment with different stimulation, investigate alternative learning paradigms, and switching off learning entirely. Such models can help us understand the relative roles of prenatal structural development and postnatal developmental plasticity.

## Methods

Our system architecture is predominantly the same as described in Adams & Harris^14^. More details of the network construction, initial parameters and neuron models are given in the Supplementary Methods. Here we restrict ourselves to summarising only the main features and new additions relevant to the current work.

### The Visual Map Architecture

Figure 1 shows the network architecture. The Input layer consists of 128 × 128 neurons and relays spike data into the network at the same resolution as the DVS camera we use to capture the moving input. The Input layer is connected to the 32 × 32 LGN layer with feed-forward excitatory connections with fixed weights of value 1.0. These connections are set up such that a 4 × 4 connection field (CF) from the Input layer is connected topologically to 1 neuron in the LGN layer (Box 1 in Fig. 1) and effects a down-sampling from 128 × 128 to 32 × 32. The ‘Cortical’ layer consists of 60 × 60 neurons of which 20% are randomly assigned as inhibitory and 80% as excitatory. The LGN and Cortical layers are not fully connected: each cortical neuron only ‘sees’ neurons from the LGN layer within a feed-forward connection field. See Box 2 in Fig. 1. The connection fields from each Cortical neuron overlap. The Cortical layer is also recurrently connected: there are sparse lateral connections and these follow a ‘mexican hat’ profile of short-range excitation and long-range inhibition. Input, LGN and Cortical neurons are modelled as spiking neurons using a simple Leaky Integrate and Fire (LIF) model.

### Input Patterns

Simplified but naturalistic visual stimulation is provided using data captured with a DVS-128 Silicon Retina Camera. The DVS camera is a specialised neuromorphic device that generates spike events in response to luminance changes in the individual pixels in the camera’s 128 × 128 array and the spikes can be directly injected to the network with minimal processing (apart from the down-sampling mentioned above). An extended bar object (as shown in Fig. 1) was moved across the camera lens in eight different directions (N, NE, E, SE, S, SW, W and NW) and the data logged in a file. Ten repeats of each direction were captured to provide variability. For more details of the DVS-128 camera and the information extraction and processing of the data it generates see References 21, 22, 23.

### Learning

Previous experimental and modelling studies have shown that Spike-Timing Dependent Plasticity (STDP) is a plausible mechanism in real neurons^24^,^25^,^26^. The relative firing times of pre- and post-synaptic neurons influence the strengthening or weakening of connections. When a pre-synaptic spike is emitted *before* a post-synaptic spike within a specified time window, there may have been a causal effect and the synaptic connection between the neurons is strengthened (Long Term Potentiation or LTP). In contrast, when the post-synaptic spike occurs first then it cannot have had a causal effect on presynaptic firing and the connection is weakened (Long Term Depression or LTD). The computational modelling study of^12^ showed how robust cortical directional selectivity could arise using STDP with an asymmetric time window combined with distance dependent delays between cortical neurons. We have adopted this method on both LGN-Cortical afferent and Cortical-Cortical lateral connections. The STDP weight change rules for the LTP and LTD elements are given as equations (1) and (2).


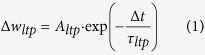



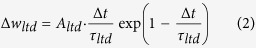


where:

Δ*w*_*ltp*_, Δ*w*_*ltd*_ are the magnitudes of the weight changes

Δ*t* is (firing time of the pre-synaptic neuron – firing time of the post-synaptic neuron)

*τ*_*ltp*_, *τ*_*ltd*_ are the LTP and LTD time constants.

*A*_*ltp*_, *A*_*ltd*_ are the LTP and LTD learning rates for STDP.

In most formulations of STDP the LTD rate (*A*_*ltd*_) is set slightly higher than the LTP rate to ensure stability^25^ and we have adopted this approach. See Table 1 for details of the parameter values we used in our learning experiments. Equations (1) and (2) result in an STDP window where the LTP part is an exponential curve as in regular STDP^25^ but the LTD part is represented by an alpha function as introduced in^12^. The effect of this asymmetric window is that LTD is deeper and persists over a longer timescale than LTP.

STDP is a form of Hebbian learning and in its standard form weights will continue to increase or decrease unbounded. Most computational studies therefore include some form of mechanism to counteract this such as global normalisation or hard limiting of synaptic weights. To avoid the need for such artificial measures we employed weight dependant updates^27^:


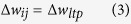



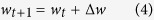



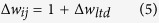



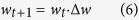


Equations (3) and (4) are the update rules for LTP. In (3), the proposed weight change for a synapse between presynaptic neuron *i* and postsynaptic neuron *j* (Δ*w*_*ij*_) takes the value Δ*w*_*ltp*_ directly from equation (1). In (4) the new weight of the synapse is calculated by adding the value from (3) to the existing weight. In (5) (LTD), (Δ*w*_*ij*_) is calculated as 1+ the value Δ*w*_*ltd*_ (from equation (2)) and in (6) the new weight of the synapse is calculated by multiplying the value from (5) by the existing weight. The effect for LTD is that Δ*w*_*ij*_ is a value less than 1.0 (as Δ*w*_*ltd*_ is always negative) and when multiplied by the existing weight results in a decrease.

### Experimental Procedure

For each experiment randomly initialised networks were generated with either the intact or disrupted connectivity and trained as follows:

- One of the eight directions (N, NE, E, SE, S, SW, W and NW) was randomly selected
- One of 10 different instances of pre-recorded data for that direction was selected
- The corresponding data file was loaded and the spike events processed to lists of x,y coordinates and timestamps
- Spike events were applied to the Input layer (128 × 128)
- Neural processing started
- Spikes were down-sampled and processed through the LGN layer (32 × 32) and propagated into the Cortical layer
- Afferent and Lateral learning (as per equations 1, 2, 3, 4) was applied continuously
- Once all spikes in the sequence had been processed, the network was reset for the next pattern (i.e. there is no interaction between successive presentations).

## Additional Information

**How to cite this article**: Adams, S. V. and Harris, C. M. A Computational Model of Innate Directional Selectivity Refined by Visual Experience. *Sci. Rep.* **5**, 12553; doi: 10.1038/srep12553 (2015).

## Supplementary Material

Supplementary Information

## Figures and Tables

**Table 1:** Summary of STDP parameters.

**Learning Rule Parameters**	**Value**
A_pa_, Afferent LTP rate	0.02
A_ma_, Afferent LTD rate	−1.05 · A_p_
A_pl_, Lateral LTP rate	0.01
A_ml_, Lateral LTD rate	−1.05 · A_p_
*τ*_*ltp*_, LTP time constant	11 ms
*τ*_*ltd*_, LTD time constant	20 ms
