# Supplementary material for: A Computational Model of Innate Directional Selectivity Refined by Visual Experience
Source: Sci Rep. 2015 Jul 31;5:12553. doi: 10.1038/srep12553 (PMC4521171; doi:10.1038/srep12553)
Supplement: Supplementary Information [file srep12553-s1.pdf]

A Computational Model of Innate Directional Selectivity Refined by Visual Experience

Samantha V. Adams*, Christopher M. Harris

Centre for Robotics and Neural Systems,
University of Plymouth, PL4 8AA Plymouth, United Kingdom

*Corresponding author. Tel.: +44 (0)1752 586289; fax: +44 (0)1752 232540.
E-mail address: samantha.adams@plymouth.ac.uk

Supplementary Methods

Here we describe in more detail the system architecture that was used in [1] and which formed the basis for the current work. New additions (for example, the plasticity mechanism) are contained in the main manuscript.

Neuron Model and Synaptic Dynamics

LGN layer neurons are modelled as simple Leaky Integrate and Fire (LIF) neurons:

$$\tau_{lm} \frac{dV_i}{dt} = -V_i \tag{S1}$$

where V_i is the membrane voltage of the LGN neuron, and τ_{lm} is the LGN neuron membrane time constant. This is essentially a simple decaying voltage affected by spikes injected from connected neurons in the Input layer. When a presynaptic (Input) neuron fires the membrane voltage, V_i of the postsynaptic (LGN) target neuron is increased via:

$$V_{1\_new} = V_{l\_\mathrm{old}} * w \tag{S2}$$

where V_{l_old} is the original membrane voltage, and V_{l_new} is the updated membrane voltage *w* is the synaptic weight. Synaptic weights were fixed at 1.0 for all connections.

To achieve a spatio-temporal down-sampling of spike input from the Input layer resolution (128x128) to the LGN layer resolution (32x32) the membrane time constant of LGN neurons (τ_{lm}) is set at 10ms and the refractory period is also 10ms. This ensures that the first firing of any Input neuron in the 4x4 group connected to the LGN neuron will cause the LGN neuron to fire but immediate firing of other Input neurons in the group within the refractory period will not cause additional spikes in the LGN neuron.

Cortical layer neurons were modelled as more complex LIF neurons, based upon the Vogels and Abbott CUBA (CUrrent BAsed) model [2]:

$$\tau_m = \frac{dV}{dt} = \left(g_e + g_i - V + N\right) \quad \text{(S3)}$$

where *V* is the membrane voltage, g_e is the contribution from excitatory synapses, g_i is the contribution from inhibitory synapses, *N* is noise (see below), and τ_m is the membrane time constant. In this model, cortical neurons receive input from both excitatory (g_e) and inhibitory (g_i) synapses which are represented by the fast AMPA (α-amino-3-hydroxy-5-methyl-4-isoxazolepropionic acid) receptor model. It is assumed that the action potential generated by the presynaptic neuron is instantaneous and decays exponentially over time [3]. A positive noise term *N(t)* was added to simulate background noise given by the 1st order system [4]:

$$\tau_n \frac{dN(t)}{dt} = -N(t) + 0.35\left[\mu_n + \sigma_n\left(\sqrt{\frac{1}{\tau_n}}\right)\eta(t)\right] \quad \text{(S4)}$$

where *N(t)* is the noise at time t, μ_n is the mean of the noise, σ_n is the standard deviation of the noise, τ_n is the time constant, and $\eta(t)$ is (standard) Gaussian white noise. Synaptic dynamics were determined by:

$$\tau_s \frac{dg}{dt} = -g \tag{S5}$$

where g is the effective conductance for an excitatory or inhibitory synapse, and τ_s is the synaptic time constant. When a presynaptic neuron fires the effective conductance for excitatory and inhibitory synapses was updated by:

$$g_{new} = g_{old} * w \tag{S6}$$

Where g_{old} is the original effective synaptic conductance, g_{new} is the updated effective synaptic conductance, and *w* is the synaptic weight. Parameter values are given in Table S1.

Network Structure Initialization

The network architecture is shown in Figure 1 of the main article. The input layer is connected to the LGN layer with excitatory connections with fixed weights of value 1.0. These connections were set up such that a 4x4 connection field (CF) from the input layer is connected topologically to 1 neuron in the LGN layer. CFs were not overlapping, thus each neuron in the LGN layer averages the activity from 16 pixels in the Input layer (the box marked 1 in Figure 1 is illustrative). The neuron time constant and refractory period for the LGN layer neurons were set to ensure that any activity in the 4x4 group of input neurons resulted in one spike in the LGN Layer neuron (i.e. no multiple firing).

Similarly, the LGN layer is not fully connected to the Cortical layer, but, in keeping with the approach of previous works modelling the visual system each cortical neuron only ‘sees’ neurons from the LGN layer within its connection field. The CFs from each cortical neuron overlap: see the box marked 2 in Figure 1 for an example. In our experiments, a 5x5 square connection field has been used as the ‘standard’ case. Afferent connection weights are set to an initial random value between 0.4 and 0.5. The Cortical layer is recurrently connected: there are sparse lateral connections and these follow a ‘mexican hat’ profile of short-range excitation and long-range inhibition. Excitatory and inhibitory connection probabilities are

determined by functions based upon distance between the two neurons as:

$$p_{exc} = \exp\left(-\frac{dist}{sigma}\right) \quad \text{(S7)}$$

$$p_{inh} = \exp\left(-\frac{sigma}{dist}\right) \quad \text{(S8)}$$

where p_{exc} is the excitatory connection probability (between 0 and 1.0), p_{inh} is the inhibitory connection probability (between 0 and 1.0), *dist* is the Euclidean distance between the neurons, and *sigma* is the spread. For our 'standard' case, Cortical excitatory connectivity uses a sigma of 3.5 which gives a significant chance of connection at distances up to 5 units. At distances greater than this the probability is forced to zero. For Cortical inhibitory connectivity a sigma of 8.0 is used and at distances less than 5 units and greater than 21 units the probability is forced to zero.

Lateral connection weights are set to an initial random value between 0.0 and 0.1. Lateral connections also incorporate delays which are calculated according to the distance between the two neurons with added Gaussian noise with mean 0 and standard deviation 0.5. Refer to Table S2 for a summary of all the network parameters and their initial values.

The network was implemented using the Brian spiking neural simulator [5].

Calculation of Neuron Preference and Selectivity Index (SI)

The average of the firing rate (number of spikes generated during presentation of a pattern) in response to each directional pattern was calculated by presenting all 10 instances of each direction to the networks and averaging the responses. This data was collected for ten untrained networks, at a point midway in training and after training. Neuron orientation and direction preference was calculated using the vector average method described in [6]. For orientation, firing rates were averaged over the two opposite directions of motion as was done in [7] and the vector sum **V**(x,y) for each neuron was calculated using equations (S9) and (S10).

$$V_x = \sum_{\phi} \eta_{\phi} \left(\cos(2 * \phi)\right) \quad \text{(S9)}$$

$$V_y = \sum_{\phi} \eta_{\phi} \left(\sin(2 * \phi) \right) \quad \text{(S10)}$$

where η_{ϕ} is the firing rate for orientation ϕ and V_x and V_y are the x and y component sums.

The preferred orientation θ can then be found using equation (S11):

$$\theta = \frac{1}{2} \operatorname{atan} 2\left(V_y, V_x\right) \quad \text{(S11)}$$

Note that equation (S11) produces orientations in the range 0 to +/- 180 degrees. To convert to 0-180 range, 180 degrees is added to negative angles.

For direction preference the same method is used except that as direction is 2π -periodic, ϕ is not multiplied by 2 in equations (S9) and (S10) and there is no division by 2 in equation (S11). Negative angles are converted to 0-360 range by adding 360 degrees.

The Selectivity Index (SI) is the magnitude of vector **V.** Normalised selectivity is calculated using equation (S12).

$$SI = \frac{\sqrt{V_x^2 + V_y^2}}{\sum_{\phi} \eta_{\phi}} \quad \text{(S12)}$$

The resulting SI takes a value between 0.0 and 1.0 with 1.0 indicating exclusive preference for one direction or orientation.

Tables

Table S1 - Summary of neuron model parameters

Parameter	Value
V_{reset}, reset voltage (LGN and cortical)	0 mV
$V_{ThreshL}$, neuron threshold (LGN)	0 mV
$V_{ThreshC}$, neuron threshold (cortical)	Randomly initialised as 1.0 mV plus noise normally distributed between 0 and 0.3 mV
τ_{lm}, membrane time constant (LGN)	10 ms
τ_m, membrane time constant (cortical)	5 ms
τ_e, excitatory synaptic time constant	5 ms
τ_i, inhibitory synaptic time constant	5 ms
τ_n, noise time constant	5 ms
μ_n, noise mean	0.7
σ_n, noise standard deviation	0.5
τ_{dl} ,delay on lateral synapses	Set as distance between pre and postsynaptic neuron plus noise added by a Gaussian with mean 0 and standard deviation 0.5
τ_{l_refrac}, neuron refractory period (LGN)	10 ms
τ_{c_refrac}, neuron refractory period (cortical)	5 ms

Table S2 - Summary of network architecture parameters

Parameter	Value
N_{in}, number of neurons in Input layer	16384 (128x128)
N_l, number of neurons in LGN layer	1024 (32x32)
N_c, number of neurons in cortical layer	3600 (60x60)
W_{aff}, afferent synaptic weights	Randomly initialised between 0.4 and 0.5
W_{lat}, lateral synaptic weights	Randomly initialised between 0.0 and 0.1 (exc) and -0.1 and 0.0 (inh)
Exc_p_{conn}, connection probability for lateral excitatory connections	Calculated as *exp(-dist/sigma)* where dist is the Euclidean distance between the neurons and sigma is 3.5
Inh_p_{conn}, connection probability for lateral inhibitory connections	Calculated as *exp(-sigma/dist)* where dist is the Euclidean distance between the neurons and sigma is 8.0

References

[1] Adams, S.V. & Harris, C. M. A proto-architecture for innate directionally selective maps, *Plos ONE*, DOI: 10.1371/journal.pone.0102908 (2014).

[2] Vogels, T.P. & Abbott, L.F. Signal propagation and logic gating in networks of integrate-and-fire neurons. *J. Neurosci*. **25**, 10786–10795 (2005).

[3] Dayan, P. & Abbott, L.F.(2001) *Theoretical Neuroscience: Computational and Mathematical Modeling of Neural Systems*. MIT Press, Cambridge Mass. (2001).

[4] Shon, A., Rao, R. & Sejnowski. T. Motion detection and prediction through spike-timing dependent plasticity. Network: Computation in Neural Systems **15**, 179-198 (2004)

[5] Goodman, D. & Brette, R. Brian: a simulator for spiking neural networks in Python, *Front Neuroinform* **2**, 1-10 (2008).

[6] Miikkulainen, R., Bednar, J., Choe, Y. & Sirosh, J. *Computational maps in the visual cortex*, Springer, New York. (2005)

[7] Ernst, U., Pawelzik, K., Sahar-Pikielny, C. & Tsodyks, M. Intracortical origin of visual maps. *Nat Rev Neurosci.* **4**, 431-436 (2001)
